# Assessing progress on the coverage of interventions in the first 1000 days in India: role of national programs

**DOI:** 10.1136/bmjgh-2024-015246

**Published:** 2024-12-20

**Authors:** Rasmi Avula, Phuong Hong Nguyen, Anita Christopher, Soyra Gune, Neena Bhatia, Alka Chauhan, L K Dwivedi, Avani Kapur, Sarang Pedgaonkar, Ritwik Shukla, Suman Chakrabarti, Shri Kant Singh, Purnima Menon

**Affiliations:** 1 Nutrition, Diet and Health Unit, International Food Policy Research Institute, Washington, District of Columbia, USA; 2 Nutrition, Diet and Health Unit, International Food Policy Research Institute, New Delhi, India; 3 Lady Irwin College, New Delhi, India; 4 International Institute of Population Sciences, Mumbai, Maharashtra, India; 5 Centre for Policy Research, New Delhi, India; 6 Center for Policy Research, Denver, Colorado, USA

**Keywords:** India, Health systems, Maternal health

## Abstract

**Background:**

High coverage of nutrition-specific interventions is critical to meet global nutrition targets, and it is imperative to understand how to attain it. We examined trends and inequalities in the coverage of interventions in India and the role of the National Nutrition Mission (NNM) in achieving improvements.

**Methods:**

We conducted trends and equity analysis of 30 interventions using two rounds of National Family Health Survey data (2015–2016, n=1 78 874, and 2019–2021, n=1 70 697). We also compared coverage between states that received incentives and monitoring under NNM and those that did not. We reviewed programme documents and grey literature to construct a policy timeline to trace pathways to coverage improvement and consulted with stakeholders to confirm interpretation of findings.

**Findings:**

Between 2016 and 2021, coverage improved significantly for nearly all interventions (~1–22 percentage points (pp) during pregnancy, ~7–20 pp during delivery/postpartum and~5–17 pp during early childhood). Improvements in coverage were higher among the poor and in rural areas compared with the rich and in urban areas, respectively; wealth and residence gaps narrowed for most interventions. These improvements could be traced to community mobilisation, technology and monitoring under NNM. Improvements in coverage of growth monitoring and counselling were higher in states that received additional incentives and monitoring under NNM. Stakeholders concurred that the improvements in coverage were likely driven by NNM.

**Interpretation:**

Focused policy attention and programmatic efforts improved coverage and reduced inequities indicating an inclusive approach. Persistent coverage gaps for certain interventions require further inquiry.

**WHAT IS ALREADY KNOWN ON THIS TOPIC**

- High coverage (>90%) of nutrition and health interventions in the first 1000 days could potentially contribute to 20% reduction in stunting. Subnational success case studies on stunting in India demonstrate that improvements in coverage of these interventions contributed to 11%–23% reduction in stunting. There is, however, a gap in understanding how such changes vary by demographic characteristics, what are the necessary conditions needed for such improvements, and what kind of policy and programmatic elements could potentially drive such changes.

**WHAT THIS STUDY ADDS**

- Our study uses nationally representative data gathered both before and after the launch of the National Nutrition Mission between 2016 and 2021. It provides an update on coverage of nutrition and health interventions across the continuum of care and examines trends in inequities by wealth. This assessment highlights areas where national nutrition programming efforts likely contributed to improved coverage. Beyond documenting changes at national level, our findings reveal that gaps in coverage narrowed the most for states that received performance-based incentives for prioritised nutrition interventions compared with those that did not receive such incentives.

**HOW THIS STUDY MIGHT AFFECT RESEARCH, PRACTICE OR POLICY**

- Our study provides evidence at scale that national policy efforts that prioritise political commitment, financing, and systems strengthening and deploy available national implementation platforms can accelerate the scale-up and uptake of nutrition and health interventions even in a short period of time. Impacts of systems strengthening efforts on coverage are facilitated by the pre-existence of implementation platforms and personnel.

## Introduction

 Nutrition, equity and inclusion feature prominently and are emphasised in the 2030 Sustainable Development agenda. The importance of a combination of nutrition-specific (eg, nutrition counselling, micronutrient supplementation) and nutrition-sensitive (eg, water, sanitation, education, poverty) interventions for tackling undernutrition and micronutrient deficiencies among women and children is well documented.^1^,^3^ A rapid scale-up of these interventions with equity and quality to all population subgroups is essential to accelerate progress toward the Sustainable Development Goals (SDG).^4^

As India is home to a quarter of world’s stunted children, achieving SDGs is contingent on its progress toward them. India has had a long history of strong stated policy intent for improving maternal and child nutrition.^5^ Several prophylactic programmes were implemented as early as in 1970s. India’s National Nutrition Policy in 1993 included goals for improving coverage of interventions through strengthening multisectoral actions for nutrition. More recently the National Nutrition Strategy reinforced a similar policy intent for reducing undernutrition by 2022.^6^

**Figure 1:**
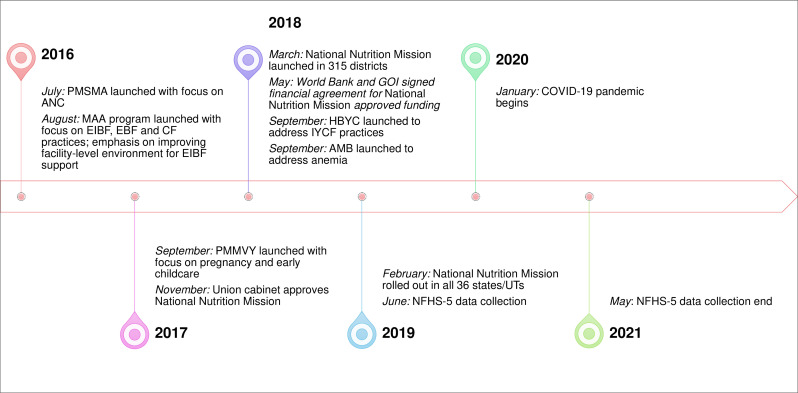
Timeline of national nutrition actions implemented between 2016 and 2021. AMB, Anemia Mukt Bharat (anaemia-free India); ANC, antenatal care; CF, complementary feeding; EBF, exclusive breastfeeding; EIBF, early initiation of breastfeeding; HBYC, home-based care for young child program; IYCF, infant and young child feeding; GOI, Government of India; NFHS, National Family Health Survey; MAA, Mother’s Absolute Affection; PMMVY, Pradhan Mantri Matru Vandana Yojana (a conditional maternity benefits program); PMSMA, Pradhan Mantri Surakshit Matritva Abhiyan (a fixed-day AMC program). UTs, Union Territories.

The Integrated Child Development Services (ICDS) programme and the National Health Mission (NHM) together have been delivering nutrition-specific interventions during the first 1000 days for more than two decades. These programmes have evolved and expanded with the goal of universal coverage. Between 2015 and 2018, multiple national programmes were initiated with an emphasis on interventions and behaviours for improving maternal and child nutrition (figure 1).

In 2018, India’s National Nutrition Mission (NNM) was launched, which brought all these actions for nutrition under one umbrella. To facilitate service delivery and improve coverage of interventions, NNM introduced technology-driven tools for frontline workers (FLWs), rejuvenated capacity building efforts, emphasised convergence among departments, focused on behaviour change communication (BCC), encouraged innovations and included grievance redressal mechanisms.^7^ The technology intervention focused on ensuring growth monitoring, timely home visits and communication of appropriate nutrition information. Community-based events, home visits and periodic campaigns focused on maternal and child nutrition were emphasised to promote behaviour change.^8^

These various efforts created an enabling environment for strengthening implementation, coordination and accountability for delivering interventions. There is, however, limited evidence on NNM’s role in improving intervention coverage and decreasing equity gaps beyond the existing national programmes. Therefore, we assessed: (1) How has the coverage of nutrition interventions changed in India between 2016 and 2021? (2) How have inequities in the coverage of nutrition interventions changed in that period? (3) What role did NNM play in improving coverage of nutrition interventions?

## Methods

We used mixed methods for the study. First, we conducted an empirical secondary data analysis to examine changes in coverage of health and nutrition interventions and inequities in coverage by wealth and place of residence between 2016 and 2021. Second, we reviewed policy documents and grey literature to construct a timeline for policy evolution. Third, we shared findings with stakeholders in the nutrition community to interpret the changes in coverage and equity.

### Data sources

The National Family Health Surveys (NFHS) follow a systematic, multi-stage stratified sampling design, covering all states in India and are representative at both state and district levels. These surveys provide comprehensive information on nutrition and health interventions. For this study, we used data from two rounds of NFHS surveys conducted in 2015–2016^9^ and 2019–2021.^10^ We focused on mother-child dyads with the youngest children, who were below 5 years of age (n=1 78 874 for 2016 and n=1 70 697 for 2021) (online supplemental figure 1). This age group was chosen as most questions on coverage are applicable to this target population.

**Figure 2:**
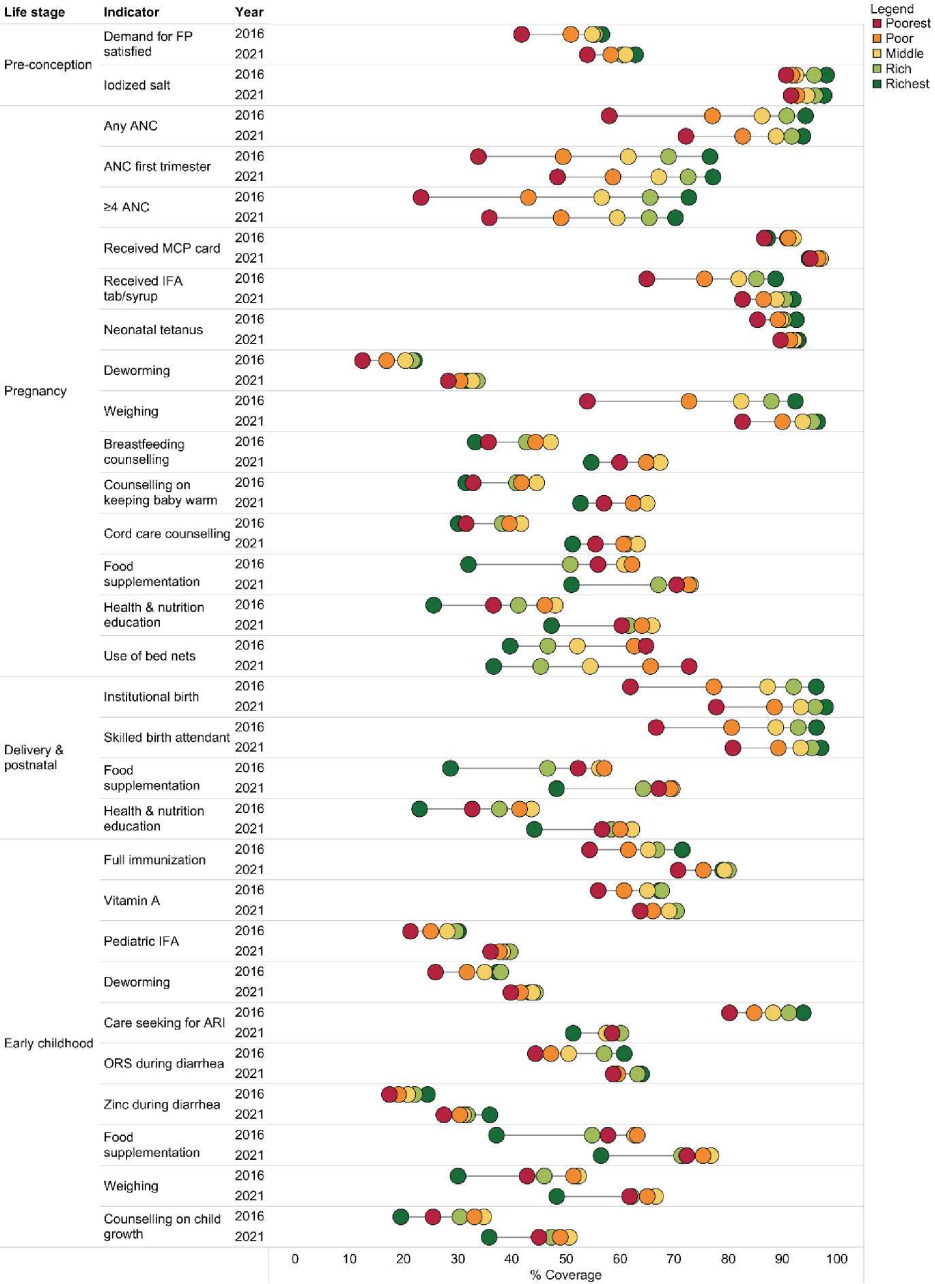
Wealth inequalities in coverage of nutrition interventions between 2015–2016 and 2019–2021. Wealth index was constructed using principal component analysis across 40 indicators of household ownership of assets and amenities. Households were then ranked from poorest to richest and divided into five quintiles.ANC, antenatal care; ARI, acute respiratory infection; FP, family planning; IFA, iron folic acid; MCP, mother-child protection; ORS, oral rehydration solution.

### List of interventions

We selected nutrition interventions based on the National Nutrition Monitoring Framework.^11^ These include: (1) *pre-pregnancy* (family planning, use of iodised salt), (2) *pregnancy* (received antenatal care (ANC), received mother-child protection (MCP) card, iron and folic acid (IFA) supplements, deworming medication, weighing, counselling (breastfeeding, cord care, keeping baby warm), food supplementation, health and nutrition education, use of bed nets), (3) *delivery and postnatal care* (institutional delivery, skilled birth attendant, food supplementation, health and nutrition education) and (4) *early childhood* (full immunisation, vitamin A, IFA supplements, deworming medication, care seeking for acute respiratory infection (ARI), oral rehydration solution (ORS) and zinc during diarrhoea, food supplementation, growth monitoring and counselling).

Indicators to assess coverage were constructed using the Demographic Health Survey or India’s programmatic guidelines, where applicable (online supplemental table 1).^12^ We excluded postnatal care for mothers and babies because comparable indicators could not be constructed for the two rounds of the NFHS, either because the questions were asked differently or ordering of the questions was changed between the rounds, which could potentially affect the responses.^13^

### Equity measures

Equity in coverage of interventions was examined using household wealth and place of residence measures. The household wealth index was constructed using 40 household-owned assets and amenities and applying principal component analysis. The first component score was used to rank the sample, and it was divided into five wealth quintiles.^14^ Place of residence included rural and urban settings. We also examined inequity by caste categories (scheduled caste/tribal, other backward classes and general).

### Geographic prioritisation

Under the NNM, 11 states (Andhra Pradesh, Bihar, Chhattisgarh, Gujarat, Jharkhand, Karnataka, Madhya Pradesh, Maharashtra, Rajasthan, Tamil Nadu, Uttar Pradesh) were designated as priority states by the Indian government. Frontline workers in these states received monthly performance-based incentives when they met certain criteria pertaining to growth monitoring, home visits and fixed-day services. These 11 states account for 82% of the total beneficiary population under the ICDS.^15^ The remaining states are labelled as non-priority states for ease of reference in this paper.

### Analysis

To examine changes in coverage of nutrition and health interventions between 2016 and 2021, we estimated weighted percentage point change at the national and state levels, using adjusted Wald test for statistical significance. All the analyses were adjusted for the cluster sampling design and sampling weights as per the DHS guidance. Furthermore, we assessed annual average rate of increase (AARI) in coverage of interventions between 2006 and 2016 and 2016 and 2021 for a select set of interventions that received additional attention under NNM to qualitatively assess if the change was beyond the expected secular trend.

To assess changes in equity in coverage, we first created equity plots for the wealth quintiles and place of residence. We then examined absolute and relative gaps by wealth for each intervention using the slope index of inequality (SII) and the concentration index (CIX), respectively.^16 17^ Both SII and CIX range from −100 to +100 where positive values represent higher coverage among the rich compared with the poor and negative values indicate higher coverage among the poor. We conducted a two-sample t-test to assess if the inequities were significantly different over time.

To examine the role of NNM in improving the coverage of interventions, we compared changes in the weighted coverage of interventions between priority and non-priority states. We shared findings with key nutrition stakeholders including those within government, non-government and research institutions, during two meetings. The first meeting was attended by eight organisations and the second by 27 institutions. Most stakeholders were not directly engaged in the implementation of NNM, but were well-informed about it, given their roles at the national and sub-national levels. The feedback from the first meeting helped us in refining the analysis. The revised findings resonated with the stakeholders in the second meeting. We undertook this process as a member-check activity to confirm the findings and to interpret improvements in the coverage of interventions, leveraging stakeholders’ insights on how things were unfolding on the ground.

We constructed a policy timeline to qualitatively trace pathways to changes in coverage of interventions. In doing so, we have identified any ANC, receipt of IFA, breastfeeding counselling during pregnancy, receipt of paediatric IFA, weighing of children and growth counselling during early childhood, as these interventions were expected to have received additional attention under NNM. We explain the results across various analyses, focusing on these interventions.

### Ethics approval

We used national level, publicly available anonymised data sets and conducted secondary data analysis.

### Patient and public involvement statement

We used a national-level, publicly available secondary dataset for the study. The participants in these national surveys (women and their children) were not involved in the design of the study and in the development of the research questions and outcome measures.

## Results

### Policy and programmatic actions

Between 2016 and 2019, programmes were introduced at the national level to support delivery of nutrition interventions during the first 1000 days (figure 1). For example, a fixed monthly ANC check-up day was started to ensure pregnant women get at least one ANC visit. Additionally, a conditional maternity benefits programme was introduced that required women to attend at least one ANC. Together, these programmes aimed at encouraging beneficiaries to use the interventions, potentially improving coverage of ANC. Other programmes such as mother’s absolute affection and home-based young-child care were introduced to strengthen BCC for infant and child feeding practices within the health system and potentially improved coverage of counselling interventions. Prior to NNM, programmes were focused on strengthening the delivery of one or two interventions, targeted at mothers or children, and were part of the ICDS or the health system. The NNM brought together all the interventions under one common mandate, focusing on both mothers and children, and was launched under the highest-level leadership of the country.

Under the NNM, new tools and mechanisms were introduced for supporting and monitoring delivery of interventions. FLWs were trained using an incremental learning approach, and an innovative mobile phone-based application was developed to aid FLWs in prioritising their daily tasks and enabling supervisors to track service delivery in real time.^18^ Guidelines for collaboration among various departmental programmes were established to ensure a cohesive approach to nutrition for mothers and children in the first 1000 days. District and subdivisional magistrates were designated to monitor implementation of the convergence mandate.^19^

Incentives were introduced to motivate states and frontline staff, rewarding them for achieving certain milestones. BCC was intensified across multiple platforms and was branded as a people’s movement.^19^ In 2018, the national anaemia campaign was also launched, complementing NNM’s efforts and emphasising provision of IFA supplements to multiple groups including pregnant women and children.,^20 21^ which could have contributed to improved coverage of IFA supplementation.

### Trends in coverage of interventions

Between 2016 and 2021, significant improvements were observed in the coverage of nearly all interventions across the continuum of care (table 1). During pregnancy, any ANC, ANC coverage in the first trimester, and four or more ANC increased by 5 percentage points (pp), 7pp and 4pp, respectively. Receipt of IFA, deworming and weighing increased by 9pp, ~13 pp and~15 pp, respectively. Counselling on various topics including breastfeeding, keeping baby warm or cord care improved by 22pp.

**Table 1:** Trends in nutrition interventions across continuum of care between 2015–2016 and 2019–2021 at national level

Intervention	2015–2016		2019–2021		Absolute change between 2019–2021 and 2015–2016	
	(n*=*1 78 874)		(n=1 70 697)			
	Percent	95% CI	Percent	95% CI	Percentage point change	95% CI
Preconception						
Demand for FP satisfied	51.8	(51.4, 52.3)	59.1	(58.7, 59.6)	7.3***	(6.6, 8.0)
Iodised salt	93.4	(93.2, 93.7)	94.3	(94.1, 94.5)	0.9***	(0.5, 1.2)
Pregnancy						
Any ANC	80.2	(79.8, 80.5)	85.3	(85.0, 85.6)	5.1***	(4.6, 5.7)
ANC first trimester	56.7	(56.3, 57.2)	64.1	(63.7, 64.5)	7.4***	(6.7, 8.1)
≥4 ANC	50.7	(50.2, 51.1)	55.2	(54.8, 55.6)	4.5***	(3.7, 5.3)
Received MCP card	89.6	(89.3, 89.9)	96.0	(95.8, 96.2)	6.4***	(6.1, 6.7)
Received IFA tab/syrup	78.5	(78.2, 78.8)	87.8	(87.5, 88.1)	9.3***	(8.8, 9.8)
Neonatal tetanus	89.1	(88.9, 89.4)	91.5	(91.3, 91.7)	2.4***	(2.0, 2.8)
Deworming	18.3	(17.9, 18.7)	31.2	(30.8, 31.6)	12.9***	(12.3, 13.5)
Weighing	76.7	(76.3, 77.0)	91.3	(91.0, 91.5)	14.6***	(14.1, 15.1)
Breastfeeding counselling	40.7	(40.3, 41.2)	62.4	(61.9, 62.8)	21.7***	(20.9, 22.4)
Counselling on keeping baby warm	38.4	(37.9, 38.8)	59.9	(59.4, 60.4)	21.5***	(20.8, 22.3)
Cord care counselling	36.3	(35.8, 36.7)	58.3	(57.9, 58.8)	22.0***	(21.3, 22.8)
Food supplementation	53.1	(52.7, 53.6)	67.2	(66.7, 67.6)	14.1***	(13.3, 14.8)
Health and nutrition education	39.9	(39.4, 40.3)	60.0	(59.5, 60.5)	20.1***	(19.4, 20.9)
Use of bed nets	53.9	(53.5, 54.3)	55.7	(55.2, 56.1)	1.8***	(0.9, 2.6)
Delivery and postnatal						
Institutional birth	81.9	(81.5, 82.2)	90.2	(90.0, 90.5)	8.3***	(7.9, 8.8)
Skilled birth attendant	84.1	(83.8, 84.4)	90.8	(90.5, 91.0)	6.7***	(6.2, 7.1)
Food supplementation	48.9	(48.4, 49.4)	64.0	(63.5, 64.5)	15.1***	(14.3, 15.9)
Health and nutrition education	36.0	(35.6, 36.4)	56.4	(55.9, 56.9)	20.4***	(19.7, 21.2)
Early childhood						
Full immunisation	63.0	(62.4, 63.7)	76.5	(75.9, 77.1)	13.5***	(12.5, 14.4)
Vitamin A	62.8	(62.3, 63.4)	67.7	(67.1, 68.2)	4.9***	(4.1, 5.6)
Paediatric IFA	26.4	(25.9, 26.9)	38.0	(37.5, 38.5)	11.6***	(10.8, 12.3)
Deworming	33.1	(32.5, 33.7)	42.4	(41.9, 43.0)	9.3***	(8.5, 10.2)
Care seeking for ARI	86.5	(85.4, 87.7)	57.4	(55.6, 59.2)	−29.1***	(-31.3, to 27.0)
ORS during diarrhoea	50.7	(49.7, 51.7)	60.4	(59.3, 61.6)	9.7***	(8.2, 11.3)
Zinc during diarrhoea	20.2	(19.4, 21.0)	30.6	(29.5, 31.7)	10.4***	(9.0, 11.8)
Food supplementation	56.0	(55.5, 56.6)	70.8	(70.2, 71.3)	14.8***	(13.9, 15.6)
Weighing	45.0	(44.5, 45.4)	60.9	(60.4, 61.4)	15.9***	(15.2, 16.7)
Counselling on child growth	28.8	(28.4, 29.3)	45.6	(45.2, 46.1)	16.8***	(16.1, 17.5)

Notes: *p<0.05, **p<0.01, ***p<0.001

ANC, antenatal care; ARI, acute respiratory infection; FP, family planning; IFA, iron folic acid; MCP, mother-child protection; ORS, oral rehydration solution.

Institutional births increased by 8pp and skilled birth attendance by ~7 pp, both attaining 91% coverage in 2021. There was a remarkable increase in the coverage of health and nutrition education (20pp) during the postnatal period.

During early childhood, coverage increased by ~5pp to ~17 pp across most interventions. Specifically, coverage of paediatric IFA increased by ~12 pp, deworming by 9pp, weighing by ~16 pp and counselling on child growth by ~17 pp. However, care seeking for ARI declined by 29pp. We found coverage to be similar for boys and girls (online supplemental table 2).

### Changes in inequities in coverage of interventions

The household, maternal and child characteristics of the study sample have remained stable between 2016 and 2021; family size declined marginally, and women’s education improved (online supplemental table 3).

Figure 2 presents changes in inequity of interventions where a dot represents coverage for a quintile, and the distance between the dots represents equity gap. The coverage of most interventions increased across quintiles, and the gap between the poorest and the richest quintiles declined between 2016 and 2021. These results are confirmed by the absolute (SII) and relative inequality (CIX) indices’ estimates (table 2).

**Table 2:** Wealth inequalities in the coverage of nutrition interventions between 2015–2016 and 2019–2021

Intervention	Year	Q1 (%)	Q5 (%)	Slope index of inequality (SII) (percent points)	Ratio (Q5:Q1)	Concentration index (CIX *100) (percent)
Preconception						
Demand for FP satisfied	2016	41.7	56.6	16.7***	1.36	5.2***
	2021	53.9	62.8	10.3***	1.17	2.7***
Iodised salt	2016	90.6	98.0	9.4***	1.08	1.6***
	2021	91.5	97.6	8.2***	1.07	1.3***
Pregnancy						
Any ANC	2016	57.9	94.2	44.2***	1.63	8.8***
	2021	72.1	93.7	28.4***	1.30	5.0***
ANC first trimester	2016	33.7	76.5	50.4***	2.27	14.9***
	2021	48.4	77.1	36.4***	1.59	9.0***
≥4 ANC	2016	23.2	72.6	57.6***	3.13	19.4***
	2021	35.8	70.1	43.0***	1.96	12.5***
Received MCP card	2016	86.5	87.2	0.9***	1.01	0.2***
	2021	95.0	94.8	0.0	1.00	0.0
Received IFA supplements	2016	64.8	88.6	28.7***	1.37	5.9***
	2021	82.5	91.9	12.0***	1.11	2.1***
Neonatal tetanus	2016	85.3	92.5	7.7***	1.08	1.4***
	2021	89.5	92.8	4.0***	1.04	0.7***
Deworming	2016	12.4	22.0	12.3***	1.77	10.8***
	2021	28.2	31.5	5.4***	1.12	2.7***
Weighing	2016	53.9	92.3	46.1***	1.71	9.8***
	2021	82.5	96.4	18.7***	1.17	3.0***
Breastfeeding counselling	2016	35.6	33.2	−1.2***	0.93	−0.5***
	2021	59.8	54.6	−4.2***	0.91	−1***
Counselling on keeping baby warm	2016	32.8	31.4	0.2	0.96	0.1
	2021	56.9	52.6	−3.3***	0.92	−0.8***
Cord care counselling	2016	31.5	30.0	−0.4	0.95	−0.1
	2021	55.4	51.1	−3.0***	0.92	−0.8***
Food supplementation	2016	55.8	31.9	−26.1***	0.57	−8.0***
	2021	70.4	51.0	−21.3***	0.72	−4.9***
Health and nutrition education	2016	36.5	25.5	−10.5***	0.70	−4.2***
	2021	60.2	47.3	−13.2***	0.79	−3.4***
Use of bed nets	2016	64.7	39.6	−31.9***	0.61	−9.6***
	2021	72.7	36.6	−45.7***	0.50	−13.3***
Delivery and postnatal						
Institutional birth	2016	61.8	96.1	42.9***	1.56	8.3***
	2021	77.7	97.9	27.9***	1.26	4.4***
Skilled birth attendant	2016	66.6	96.2	37.3***	1.44	6.9***
	2021	80.7	97.0	22.0***	1.20	3.5***
Food supplementation	2016	52.2	28.6	−25.5***	0.55	−8.5***
	2021	67.0	48.2	−20.4***	0.72	−5.0***
Health and nutrition education	2016	32.6	22.9	−8.8***	0.70	−3.9***
	2021	56.6	44.1	−12.3***	0.78	−3.4***
Early childhood						
Full immunisation	2016	54.3	71.4	19.6***	1.31	5.0***
	2021	70.6	78.8	11.6***	1.12	2.4***
Vitamin A	2016	55.9	67.2	15.2***	1.20	3.9***
	2021	63.6	70.3	9.4***	1.11	2.2***
Paediatric IFA	2016	21.2	30.1	11.5***	1.42	7.0***
	2021	36.0	38.9	4.1***	1.08	1.7***
Deworming	2016	25.8	37.3	15.0***	1.45	7.3***
	2021	39.7	43.3	5.3***	1.09	2.0***
Care seeking for ARI	2016	80.1	93.7	16.9***	1.17	3.1***
	2021	58.4	51.3	−5.3	0.88	−1.4*
ORS during diarrhoea	2016	44.3	60.7	20.1***	1.37	6.3***
	2021	58.6	63.9	6.6***	1.09	1.7***
Zinc during diarrhoea	2016	17.3	24.4	8.2***	1.41	6.4***
	2021	27.4	35.9	8.7***	1.31	4.4***
Food supplementation	2016	57.7	37.1	−20.6***	0.64	−5.9***
	2021	72.2	56.4	−16.5***	0.78	−3.6***
Weighing	2016	42.8	30.0	−12.6***	0.70	−4.5***
	2021	61.7	48.2	−14.0***	0.78	−3.6***
Counselling on child growth	2016	25.4	19.4	−5.1***	0.76	−2.8***
	2021	44.9	35.7	−9.1***	0.80	−3.1***

Note 1: Significant difference (*p<0.05, **p<0.01 and ***p<0.001).

Note 2: All SII and CIX coefficients are significantly different (p<0.05) between 2016 and 2021 using the two-sample t test.

Note 3: The wealth index was constructed using principal component analysis across 40 indicators of household ownership of assets and amenities. Households were then ranked from poorest to richest and divided into five quintiles.

ANC, antenatal care; ARI, acute respiratory infection; FP, family planning; IFA, iron folic acid; MCP, mother-child protection; ORS, oral rehydration solution; Q1, wealth quintile 1; W5, wealth quintile 5.

The gap between the richest and the poorest quintiles (Q5–Q1) narrowed for ANC, IFA and weighing and was driven by improvements in the coverage in Q1. The gap between Q1 and Q5 increased marginally in favour of Q1 for counselling on breastfeeding, keeping baby warm and cord care. The absolute gap narrowed for institutional delivery because of higher improvements in coverage in Q1 compared with Q5. For most interventions during early childhood, the absolute gap declined, including immunisation (17pp to 8 pp), vitamin A (11pp to 6 pp), IFA (8pp to 2 pp), deworming (11pp to 3pp) and care seeking for ARI (13pp to −7 pp). Coverage of weighing and growth counselling interventions continued in favour of Q1. For food supplementation and health and nutrition education interventions across the continuum of care, the gap declined marginally in favour of Q5.

Gaps in coverage of interventions based on whether mothers and children lived in rural or urban areas have decreased for nearly all interventions (online supplemental figure 2). Both in 2016 and 2021, coverage was higher in urban compared with rural areas for ANC, receipt of IFA supplementation, deworming, weighing, institutional delivery and skilled birth attendant. It, however, narrowed in 2021 due to greater improvements in coverage in rural areas compared with urban areas. Coverage was higher in rural compared with urban areas in 2016 for counselling on breastfeeding (43.9% vs 33.4%) and cord care (38.9% vs 30.1%), health and nutrition education (44.0% vs 30.8%), and food supplementation (60.1% vs 36.9%). This pattern continued in 2021, but coverage increased in urban areas, and the gap narrowed. Food supplementation and health and nutrition education coverage during postnatal and early childhood periods followed similar trends. Gaps in the coverage of paediatric IFA and deworming during early childhood were marginal, while coverage of weighing children and growth counselling remained high in rural areas. Changes in coverage of nutrition and health interventions by caste are presented in the online supplemental figure 3.

### Role of National Nutrition Mission (NNM) in improving coverage of interventions

**Figure 3:**
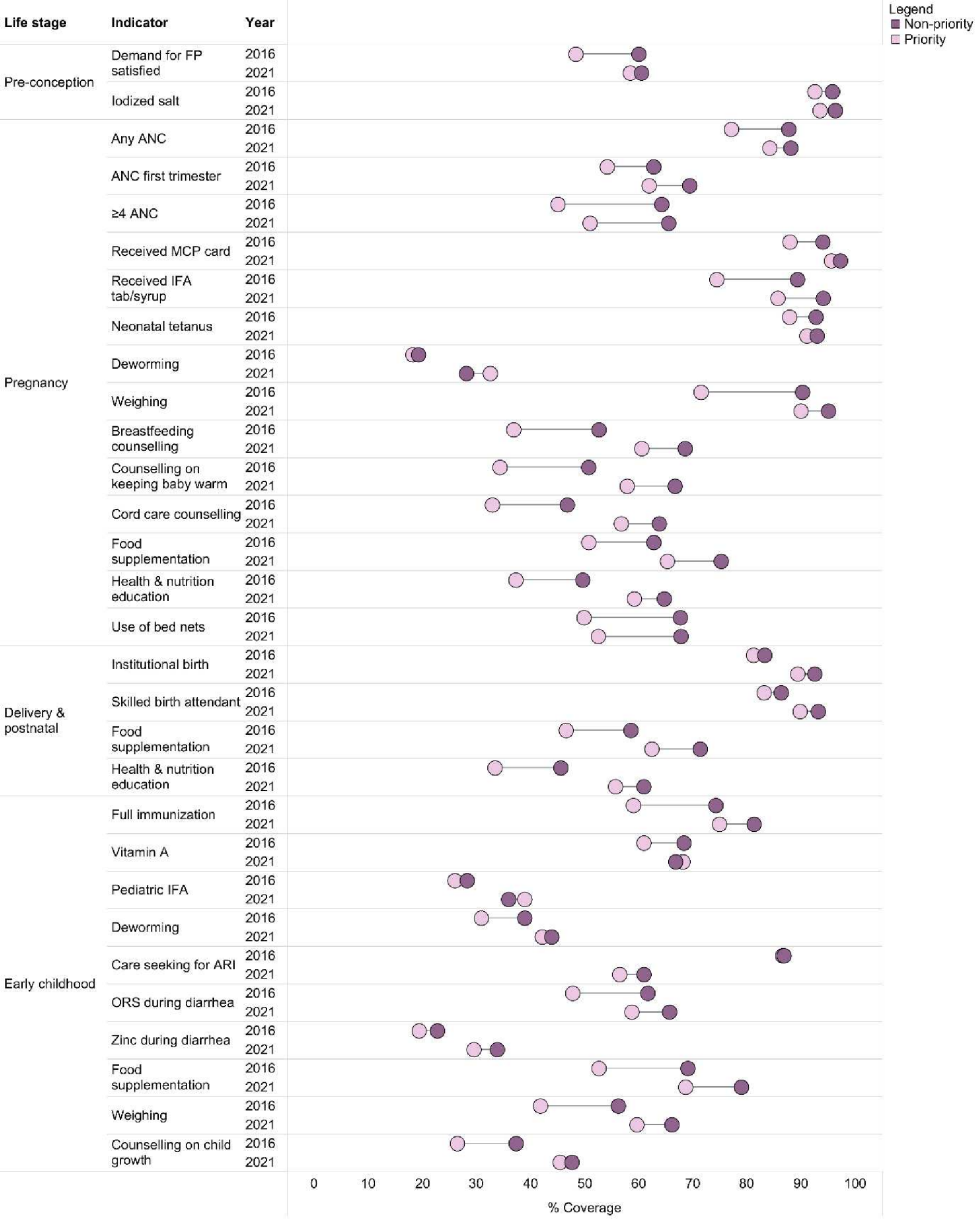
Differences between priority states and non-priority states in the coverage of nutrition interventions across continuum of care between 2015–2016 and 2019–2021. ANC, antenatal care; ARI, acute respiratory infection; FP, family planning; IFA, iron folic acid; MCP, mother-child protection; ORS, oral rehydration solution.

In 2016, coverage was lower among priority states compared with non-priority states. Between 2016 and 2021, there was significantly higher improvement in coverage across most interventions among the priority compared with the non-priority states, thus narrowing the gap between them (figure 3).

Coverage of any ANC, ANC in the first trimester and at least four ANC improved by 5pp to 7pp in the priority states compared with only 0.3pp to 6pp in the non-priority states (online supplemental table 4). Coverage for IFA, deworming and weighing improved remarkably by 11pp, 14pp and 18pp, respectively, in the priority states, compared with only 4pp to 8pp in the non-priority states. Similar changes were observed for counselling and food supplementation. The change in coverage in institutional birth was slightly lower in priority (8pp) compared with non-priority states (9pp), but similar for skilled birth attendance. Coverage for immunisation, vitamin A, IFA, deworming, weighing and growth counselling improved significantly by 15pp, 7pp, 12pp, 11pp, 17pp and 19pp among the priority states and was higher compared with the non-priority states. We find that AARI for coverage of ANC, IFA supplementation, counselling on breastfeeding, paediatric IFA, growth monitoring and counselling on child growth was higher for the non-priority states compared with the priority states between 2006 and 2016. But, between 2016 and 2021, AARI was higher for the priority states compared with the non-priority states (online supplemental figure 4).

These improvements in coverage of interventions among the priority states could be traced to NNM and additional incentivisation for the 11 states, which created an enabling environment for strengthening implementation. Various individual-intervention focused programmes were brought under the umbrella of NNM (online supplemental table 5). ANC and counselling during pregnancy and IFA supplementation received focus among the priority states under NNM. Growth monitoring was a prioritised intervention that received technological support and additional incentives in priority states to improve its coverage, along with monitoring. FLWs received smartphones with an application to support growth monitoring and counselling and home visits.^22^ Community-based events and door-to-door campaigns were organised to raise awareness for behaviour change. Monitoring mechanisms coupled with incentives were established to ensure delivery of interventions in the 11 priority states. These activities could have contributed to improving health and nutrition education interventions. State Nutrition Resource Centers were encouraged to be established so that implementation of the NNM could be monitored and six of the 11 priority states had set these up.^23^ The nutrition stakeholders, who had observed NNM’s implementation in the communities, had confirmed potential linkages between improved coverage and NNM.

## Discussion

Our study shows definitive improvements in the coverage of nutrition and health interventions between 2016 and 2021 across the continuum of care. Inequity by wealth and residence declined for most interventions. However, inequity continued to exist primarily for interventions during pregnancy. For food supplementation, growth monitoring and various counselling interventions, the poorest had higher coverage compared with the richest. Any ANC, IFA supplementation, growth monitoring and counselling were prioritised interventions under the NNM and changes in their coverage indicate that the policy and programmatic focus did have an impact on their implementation. This is further corroborated by increased coverage among the priority states that received incentives under NNM.

Inequalities in coverage of ANC are driven by several factors including wealth and residence. Similar gaps by wealth and residence, despite improvements, were observed in other settings as well.^24^,^26^ A multicountry analysis indicates women from wealthier families, those with higher education and with higher empowerment have access to quality ANC compared with those who do not.^27^ Decline in inequities in coverage could have been due to overall greater focus on expanding the reach of the programmes. There have been marked changes in the inequality patterns for any ANC and ANC in the first trimester, but not as much change in at least four ANC. These changes could have been due to renewed emphasis on the fixed-day ANC public-private partnership programme to ensure ANC for all pregnant women.^28^ As the programme did not focus on achieving a specific number of ANC, there remained a large gap for at least four ANC, between the highest and the lowest wealth quintiles. Community-based interventions or health system interventions have been shown only to marginally improve coverage of at least four ANC.^29^ The focus of India’s maternity benefits programme on early registration, and this could have acted as a gateway to increase ANC coverage. Other studies on maternity benefits have indicated increase in the utilisation of ANC.^30 31^ A summary of reviews from low- and middle-income countries supports these findings of financial incentives increasing maternal service utilisation.^32^ Improvements in equity for IFA supplementation could be attributed to the anemia-free India campaign and to intensify BCC for changes in counselling and growth monitoring interventions. Although there has been strong progress, these interventions are yet to achieve universal coverage that is essential for improving nutrition outcomes. It is also imperative to ensure quality of interventions along with the reach.^4^

Technological intervention combined with monitoring mechanisms could have facilitated improvements in growth monitoring and counselling. Enabling FLWs with digital technologies has improved growth monitoring, counselling and timely home visits, particularly in places where the coverage was low.^33^ FLWs preferred using the mobile-based application as growth charts were generated automatically, an in-built planner helped in prioritising home visits and digital materials helped in counselling.^18^ Literature shows that digital innovations improve FLW service delivery, and job aids facilitate in improving child feeding practices.^34 35^ The community-based events and home visits were identified to be important platforms for effective BCC.^36^ Community-based events were routinised and awareness campaigns were promoted, and these were all implemented with the participation of staff from multiple sectors and millions of people between September 2018 and April 2022.^37^,^39^

Under NNM, 18 departments pertaining to nutrition were mandated to work together.^37^,^39^ Supra-departmental authorities were designated to ensure convergence at various levels of implementation, moving away from the usual practice of relying solely on departmental leads. Such a change likely helped navigate interdepartmental complexities such as bureaucratic hierarchies or competing interests that can impede collaboration.^40^ Additionally, community events, designated weeks for nutrition activities and increased emphasis on home visits under NNM facilitated multiple interactions between FLWs and beneficiaries. Together, these efforts renewed focus on mothers and children below 2 years of age.

Multiple platforms were used to ensure delivery of interventions. For example, anemia-free India campaign was launched, which focused on increased coverage of IFA supplements along with BCC, and test and treat approaches.^21^ Technology, training and incentives were introduced to improve service delivery. Financial incentives along with monitoring mechanisms bolstered implementation that reflected in the improved coverage of interventions in the priority states that were lagging in 2016.^23^

From a fiscal lens, these new interventions have not led to a significant rise in budgets for nutrition. Budgets for existing programmes such as the ICDS and the Reproductive and Child Health under the NHM have stagnated. Under the NNM, fund utilisation was low; only 66% of the released funds were spent. Budget was increased only for the anemia-free India campaign.^41^ It is plausible that the existing resources were efficiently used, contributing to the improvements in coverage and equity. For instance, creation of national councils spanning multiple ministries allowed for moving away from a completely siloed approach towards more coordinated action.^42^ At the district and block level, the leadership of the District Magistrate and Sub-Divisional Magistrate has been useful in chairing convergence action plan meetings and bringing departments together.^43^ Subnational level performance on implementation of NNM was monitored centrally by ranking the states on governance and institutional mechanisms, strategy and planning, service delivery and intervention coverage.^44^ Overall, monitoring by the highest leadership, support from multiple partners, availability of financially resources, had facilitated improvements in coverage of interventions. These are the key ingredients for scaling up of interventions.^45^ While continuing the trajectory of achieving universal coverage, there is a need to start establishing national guidance for improving quality of the interventions, such that the competence of providers and experiences of clients improves.^46^ In parallel, investments are needed to reduce gaps in human resources and to improve the quality of staff experiences and motivation while delivering services.

The study is not longitudinal at the individual level, but instead, interpretations are available at the state and national levels, which can act as an approximation of a snapshot of the population in an area. While the NFHS has rich data on nutrition and health interventions, it does not provide data for all interventions under the NNM. This is largely a descriptive study documenting changes in coverage. However, it has identified elements of programmes and conditions of an enabling environment that drove the changes. Although we did not consult the programme beneficiaries under the NNM to confirm the study interpretations, we did share them with stakeholders at the national level.

## Conclusion

Our study provides evidence that at-scale implementation of interventions is feasible when there is high level political commitment, sharpened focus on how to deliver the interventions, clear guidance, and accountability along with incentives. Increasing overall coverage facilitated reduction in inequalities as well.

Further research is required to understand the reasons for persistent coverage gaps for some interventions. Particularly, interventions such as at least four ANC visits, paediatric IFA supplementation and counselling for behaviour change need special attention. ANC visits act as a gateway for overall well-being check-ups of pregnant women and lay the foundation for maternal and child health at birth and beyond. High rates of anaemia among 6–59 months-old children warrant urgent actions to ensure paediatric IFA reaches all children. Counselling during various life stages continues to be important and relevant for improving maternal and child health nutrition. Therefore, there is a need for a combination of investments in implementation and in research to improve the coverage with quality and equity.

## Supplementary material

10.1136/bmjgh-2024-015246 online supplemental file 1

## Data Availability

Data are available in a public, open access repository. Data are available upon reasonable request.
