# Supplementary material for: Assessing progress on the coverage of interventions in the first 1000 days in India: role of national programs
Source: BMJ Glob Health. 2024 Dec 20;9(12):e015246. doi: 10.1136/bmjgh-2024-015246 (PMC11667467; doi:10.1136/bmjgh-2024-015246)
Supplement: online supplemental file 1 [file bmjgh-9-12-s001.pdf]

Supplemental material for "Assessing progress on the coverage of interventions in the first 1,000 days in India: Role of national programs"

Contents

Supplemental Figure 1. Analytic sample used to examine trends in coverage and inequities of health and nutrition interventions 2

Supplemental Figure 2. Residential inequalities in coverage of nutrition interventions across continuum of care between 2015-16 and 2019-21 3

Supplemental Figure 3: Caste-based differences in the coverage of nutrition interventions across continuum between 2015-16 and 2019-21 4

Supplemental Table 1: Definitions of indicators for measuring the coverage of health and nutrition interventions during the first 1,000 days 5

Supplemental Table 2: Trends in childhood interventions by sex 8

Supplemental Table 3: Summary statistics of the study sample, by survey year 9

Supplemental Table 4: Trends in coverage of nutrition interventions in priority – and non-priority states between 2015-16 and 2019-21 10

Supplemental Table 5: Interventions that were prioritized or received focus from national programs 11

Supplemental Figure 4: Annual average rate of increase in coverage of interventions, by priority and non-priority states between 2005-06 and 2015-16 and 2019-21 13

Web appendix 1: Construction of wealth index and quintiles 14

Supplemental Figure 1. Analytic sample used to examine trends in coverage and inequities of health and nutrition interventions

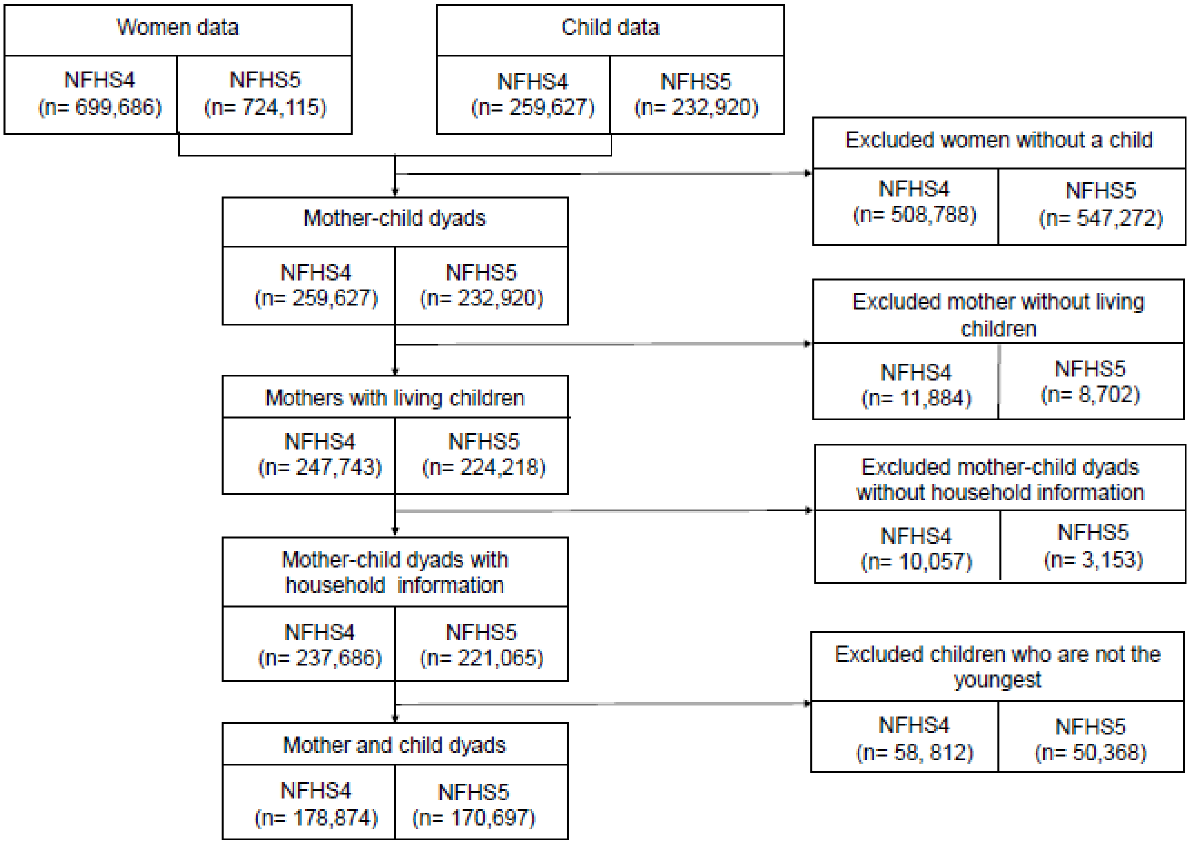

Note 1: Sample size is further restricted for the following interventions: Demand for family planning satisfied and iodized salt during pre-pregnancy, received mother-child protecion card during pregnancy, food supplementation and health and nutrition education during lactation, and full immunization, vitamin A, pediatric iron-folic acid, deworming, oral rehydration salts during diarrhea, zinc during diarrhea, and food supplementation during early childhood.

Supplemental Figure 2. Residential inequalities in coverage of nutrition interventions across continuum of care between 2015-16 and 2019-21

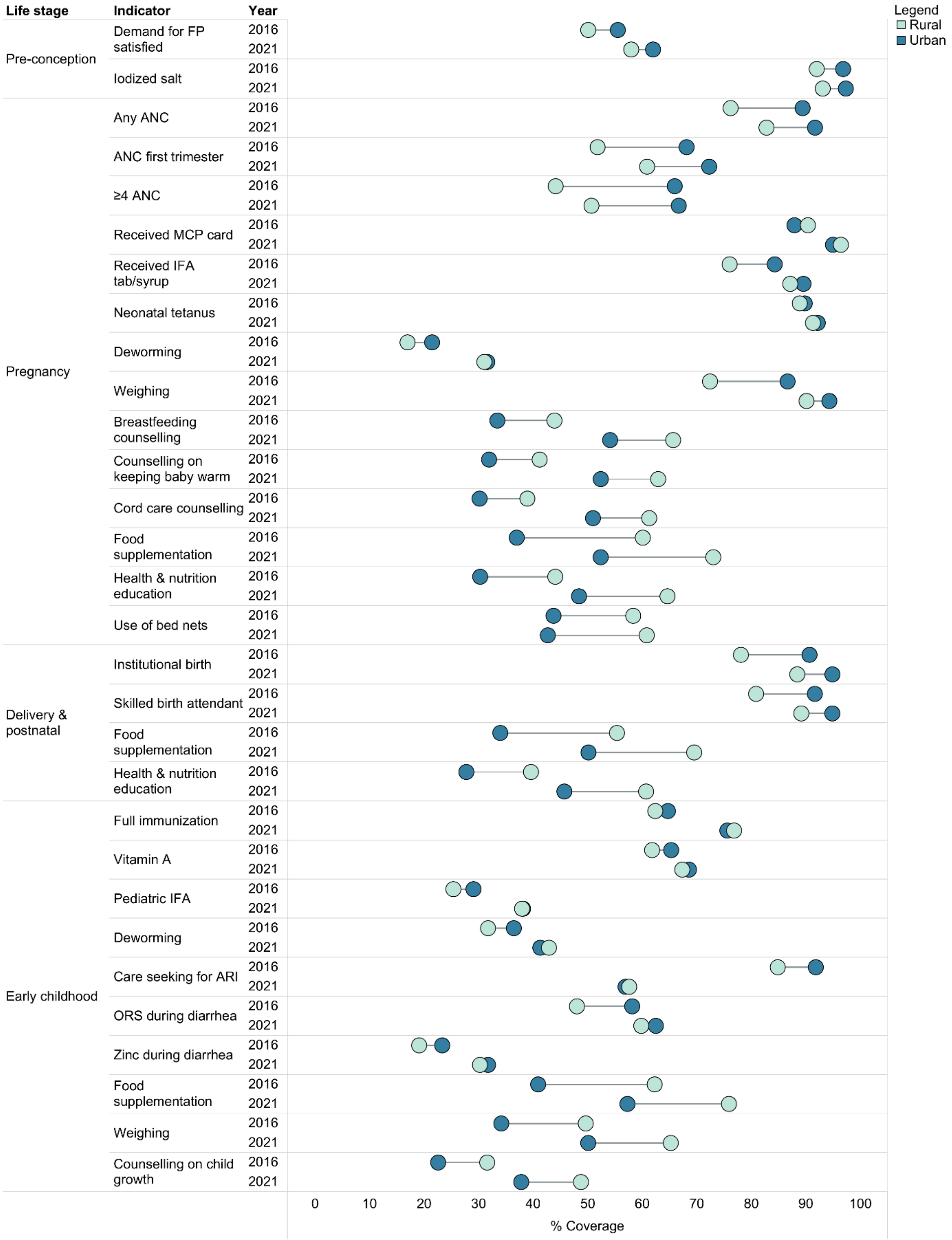

FP: Family planning, ANC: Antenatal care, MCP: Mother child protection, IFA: Iron folic acid

Supplemental Figure 3: Caste-based differences in the coverage of nutrition interventions across continuum between 2015-16 and 2019-21

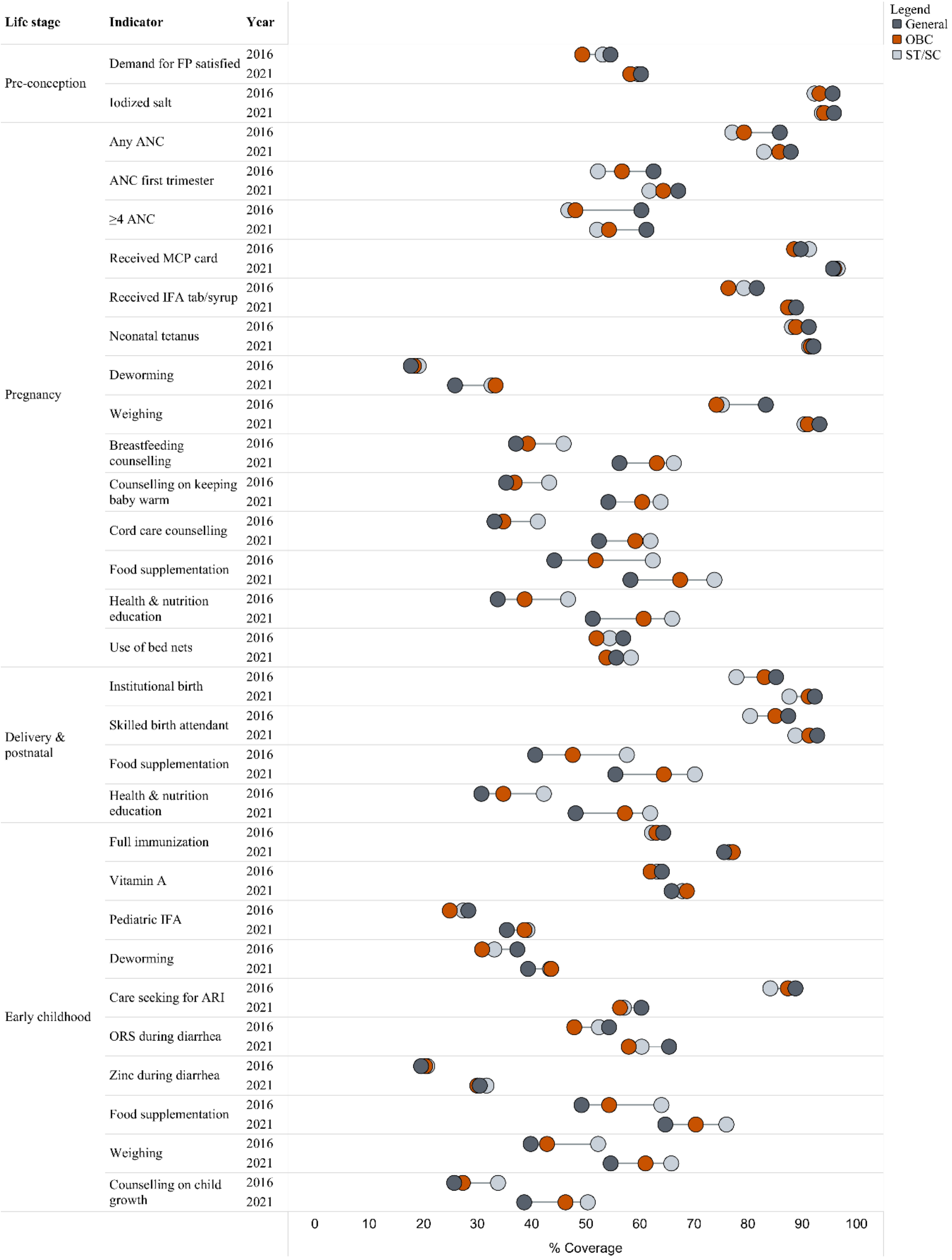

FP: Family planning ANC: Antenatal care MCP: Mother child protection IFA: Iron folic acid ICDS: Integrated Child Development Scheme. Sample for ORS and zinc during diarrhea is children below five years who had diarrhea in the two weeks preceding the survey.

Supplemental Table 1: Definitions of indicators for measuring the coverage of health and nutrition interventions during the first 1,000 days

Indicators	Definition	Numerator	Denominator
**Pre-pregnancy**			
Demand for FP satisfied	Percentage of women aged 15-49 years with youngest child below five years with demand for family planning satisfied by modern methods.	Number of women using any modern contraceptive method.	Number of women 15-49 years with youngest child below five years that have either met or unmet need for family planning.
Iodized salt	Percentage of households with youngest child below five years with iodized salt.	Number of households with iodized salt	Number of households with youngest child below five years with salt tested
**Pregnancy**			
Any ANC	Percentage of women aged 15-49 years who received antenatal care from any skilled provider during pregnancy for the most recent live birth in the five years preceding the survey.	Number of women who received antenatal care from a skilled provider for the most recent birth	Number of women aged 15-49 years with a birth in the five years preceding the survey
ANC first trimester*	Percentage of women aged 15-49 years who received antenatal care from a skilled provider during the first for the most recent birth in the five years preceding the survey.	Number of women who received antenatal care from a skilled provider during the first trimester	Number of women aged 15-49 years who had the most recent birth in the five years preceding the survey
≥4 ANC*	Percentage of women aged 15-49 years who received 4 or more antenatal care visits from a skilled provider for the most recent birth in the five years preceding the survey.	Number of women who had 4 or more antenatal care visits from a skilled provider	Number of women aged 15-49 years who had the most recent birth in the five years preceding the survey
Received MCP card*	Percentage of women aged 15-49 years who received mother and child protection card (MCP) during pregnancy for the most recent birth in the five years preceding the survey	Number of women who received MCP card during pregnancy	Number of women aged 15-49 years who had the most recent birth in the five years preceding the survey
Received IFA tab/syrup	Percentage of women aged 15-49 years who received IFA (given or purchased) during pregnancy for the most recent live birth in the five years preceding the survey	Number of women who received IFA (given or purchased) during pregnancy	Number of women aged 15-49 years who had the most recent live birth in the five years preceding the survey
Neonatal tetanus	Percentage of women aged 15-49 years who received two or more tetanus injections for the most recent birth in the five years preceding the survey	Number of women who received two or more tetanus injections during pregnancy	Number of women aged 15-49 years who had the most recent birth in the five years preceding the survey
Deworming	Percentage of women aged 15-49 years who took deworming medication during pregnancy for the most recent live birth in the five years preceding the survey.	Number of women who took deworming medication during pregnancy	Number of women aged 15-49 years who had the most recent birth in the five years preceding the survey
Weighing*	Percentage of women aged 15-49 who were weighed during pregnancy for the most recent birth in the five years preceding the survey	Number of women who were weighed during pregnancy	Number of women aged 15-49 years who had the most recent birth in the five years preceding the survey

Indicators	Definition	Numerator	Denominator
Breastfeeding counselling*	Percentage of women aged 15-49 years who met with a community health worker in the last three months of pregnancy for the most recent birth in five years preceding the survey and were advised on breastfeeding.	Number of women who received advice on breastfeeding from a community health worker in the last three months of pregnancy	Number of women aged 15-49 years who had the most recent birth in the five years preceding the survey
Counselling on keeping baby warm*	Percentage of women aged 15-49 years who met with a community health worker in the last three months of pregnancy for the most recent birth in five years preceding the survey and were advised on keeping the baby warm.	Number of women who received advice on keeping baby warm from a community health worker in the last three months of pregnancy	Number of women aged 15-49 years who had their last birth in the five years preceding the survey
Cord care counselling*	Percentage of women aged 15-49 years who met with a community health worker in the last three months of pregnancy for the most recent birth in five years preceding the survey and were advised on cord care.	Number of women who received advice on cord care from a community health worker in the last three months of pregnancy	Number of women aged 15-49 years who had their last birth in the five years preceding the survey
Food supplementation*	Percentage of women aged 15-49 years with youngest child below five years who received food supplements from ICDS during pregnancy.	Number of women who received food supplements from ICDS during pregnancy	Number of women aged 15-49 years with youngest child below five years
Health & nutrition education*	Percentage of women aged 15-49 years with youngest child below five years who received health and nutrition education from ICDS during pregnancy	Number of women who received health and nutrition education from ICDS during pregnancy	Number of women aged 15-49 years with youngest child below five years
Use of bed nets*	Percentage of women aged 15-49 years who slept under a treated bed net during pregnancy of the most recent live birth in the five years preceding the survey.	Number of women who slept under a treated bed net during pregnancy	Number of women aged 15-49 years who had the most recent live birth in the five years preceding the survey.
**Delivery & postnatal**			
Institutional birth	Percentage of live births in the five years preceding the survey to women aged 15-49 years in a health facility for the most recent live birth	Number of live births delivered in a health facility	Number of recent live births in the five years preceding the survey to women aged 15-49 years
Skilled birth attendant	Percentage of live births years in the five years preceding the survey to women aged 15-49 that were assisted by a skilled provider.	Number of live births that were assisted by a skilled provider	Number of recent live births in the five years preceding the survey to women aged 15-49 years
Food supplementation*	Percentage of women aged 15-49 years with youngest child under five years who received supplementary food from ICDS during breastfeeding.	Number of women who received supplementary food from ICDS during breastfeeding	Number of women aged 15-49 years with youngest child under five years
Health & nutrition education*	Percentage of women aged 15-49 years with youngest child under five years who received health and nutrition education from ICDS during breastfeeding	Number of women who received health and nutrition education from ICDS during breastfeeding	Number of women aged 15-49 years with youngest child under five years

Indicators	Definition	Numerator	Denominator
**Early childhood**			
Full immunization	Percentage of children aged 12-23 months who received all basic vaccines at any time before the survey according to either the vaccination card or mother's report.	Number of children who received all basic vaccines at any time before the survey according to either the vaccination card or mother's report	Number of living children aged 12-23 months
Vitamin A*	Percentage of children aged 9-35 months who received vitamin A supplements in the six months preceding the survey	Number of children who received vitamin A supplements in the six months preceding the survey	Number of living children aged 9-35 months
Pediatric IFA*	Percentage of children aged 6-36 months who were given iron supplements in the seven days preceding the survey	Number of children who were given iron supplements in theseven days preceding the survey	Number of living children aged 6-36 months
Deworming*	Percentage of children aged 12-36 months who received deworming medication in the six months preceding the survey	Number of children who received deworming medication in the six months preceding the survey	Number of living children aged 12-36 months
Care seeking for ARI	Percentage of children under age 5 with symptoms of ARI at any time in the 2 weeks preceding the survey for whom advice or treatment was sought	Number of living children under age 5 with symptoms of ARI in the 2 weeks preceding the survey for whom treatment was sought excluding advice or treatment from traditional practitioners	Number of children under age 5 with symptoms of ARI in the 2 weeks preceding the survey
ORS during diarrhea	Percentage of living children below five years with diarrhea who were given fluid made from oral rehydration salts (ORS) or pre-packaged ORS fluid.	Number of children who were given fluid made from oral rehydration salts (ORS) or pre-packaged ORS fluid	Number of living children below five years with diarrhea in the two weeks preceding the survey
Zinc during diarrhea	Percentage of living children below five years with diarrhea who were given zinc	Number of children who were given zinc	Number of living children below five years with diarrhea in the two weeks preceding the survey
Food supplementation*	Percentage of children aged 6-35 months who received food supplements from ICDS in the twelve months preceding the survey.	Number of children who received food supplements from ICDS	Number of children aged 6-35 months
Weighing*	Percentage of children below five years who were weighed at an anganwandi centre (AWC) or ICDS centre in the twelve months preceding the survey.	Number of children who were weighed at an AWC or ICDS centre	Number of children below five years
Counselling on child growth*	Percentage of mothers with children below five years who received counselling on child growth from an ICDS/anganwadi worker or auxillary nurse midwife (ANM) at an AWC in the twelve months preceding the survey.	Number of mothers who received counselling on child growth from an ICDS/anganwadi worker or ANM	Number of women with children below five years

Note: Indicator definitions are based on the Demographic Health Survey guide; *Indicator definitions are based on India's programmatic guidance

Supplemental Table 2: Trends in childhood interventions by sex

	**2015-2016**			**2019-2021**		
	Female	Male	Difference	Female	Male	Difference
Full immunization (12-23m)	63.4	62.7	-0.7	76.0	76.9	0.9
Vitamin A (9-35m)	62.6	63.0	0.4	67.8	67.5	-0.3
Pediatric IFA (6-36m)	25.9	26.9*	1.0	38.0	38.0	0.1
Deworming (12-36m)	32.7	33.5	0.8	42.6	42.3	-0.3
Care seeking for ARI	84.1	88.3***	4.2	55.8	58.7*	2.9
ORS during diarrhea	49.8	51.4*	1.6	59.1	61.5	2.5
Zinc during diarrhea	19.8	20.5	0.7	30.3	30.8	0.6
Food supplementation (6-35m)	56.2	55.9	-0.3	71.0	70.6	-0.4
Weighing	45.6	44.4***	-1.2	61.5	60.4	-1.0
Counselling on child growth	29.6	28.2***	-1.4	46.4	45.0	-1.3

*** Significant difference between males and females. *p<0.05, **p<0.01, ***p < 0.001.

IFA: Iron folic acid; ARI: Acute respiratory infection; ORS: Oral rehydration salts

Note: Sample for ORS during diarrhea and zinc during diarrhea includes only children below five years who had diarrhea in the two weeks preceding the survey. Sample for careseeking for ARI includes only children below five years who had symptoms of ARI in the two weeks preceding the survey.

Supplemental Table 3: Summary statistics of the study sample, by survey year

	2015-2016 (*n*= 178,874*)*		2019-2021 (*n*= 170,697)	
**Characteristics**	**Mean/%**	**95% CI**	**Mean/%**	**95% CI**
**Household**				
Family size, *n*	6.1	[6.1, 6.1]	6.0	[6.0, 6.0]
Wealth index[1], *1-5*				
Quintile 1 (Poorest)	22.1	[21.8, 22.5]	21.8	[21.4, 22.1]
Quintile 2	21.5	[21.1, 21.8]	20.9	[20.5, 21.2]
Quintile 3	20.1	[19.8, 20.5]	19.6	[19.3, 19.9]
Quintile 4	19.3	[18.9, 19.7]	19.6	[19.2, 20.0]
Quintile 5 (Richest)	17.0	[16.6, 17.4]	18.2	[17.8, 18.5]
Religion, *%*				
Hindu	78.8	[78.3, 79.4]	79.6	[79.1, 80.1]
Muslim	16.1	[15.5, 16.6]	15.9	[15.4, 16.4]
Christian	2.2	[2.0, 2.3]	2.1	[2.0, 2.2]
Caste categories, *%*	3.0	[2.8, 3.2]	2.4	[2.3, 2.5]
Scheduled castes				
Scheduled tribe	21.0	[20.6, 21.4]	22.5	[22.1, 23]
Other backward classes	10.2	[9.9, 10.5]	9.8	[9.6, 10.1]
Urban residence, *%*	43.5	[43.0, 44.0]	43.0	[42.5, 43.5]
Health insurance, %	24.2	[23.9, 24.6]	36.6	[36.2, 37.0]
**Mother**				
Age, *y*	26.9	[26.8, 26.9]	27.1	[27.1, 27.2]
Education[2], *%*				
No schooling	26.7	[26.3, 27.0]	19.3	[19.0, 19.7]
Primary school	13.2	[13.0, 13.4]	11.5	[11.3, 11.8]
Upper Primary school	17.8	[17.5, 18.1]	17.1	[16.8, 17.4]
Secondary school	18.9	[18.6, 19.2]	20.2	[19.9, 20.5]
Senior secondary	11.1	[10.8, 11.3]	14.3	[14.1, 14.5]
College or higher	12.3	[12.0, 12.6]	17.5	[17.1, 17.9]
**Child**				
Gender (female), *%*	45.5	[45.2, 45.8]	46.2	[45.8, 46.5]
Age, *months*	25.4	[25.3, 25.5]	26.1	[26.0, 26.2]
Birth order				
First	34.0	[33.7, 34.4]	34.4	[34.0, 34.7]
Second	35.0	[34.7, 35.4]	36.6	[36.3, 36.9]
Third or more	31.0	[30.6, 31.3]	29.0	[28.7, 29.4]

[1]The wealth index was constructed using principal component analysis and included 40 indicators of household ownership of assets and amenities. [2]Education categories were grouped for total number of years of education attainted by the individual as follows: No schooling=0 years, Primary school=1-5 years, Upper primary school=6-8 years, Secondary school=9-10 years High school=11-12 years, College or higher >12 years. CI: confidence interval

Supplemental Table 4: Trends in coverage of nutrition interventions in priority – and non-priority states between 2015-16 and 2019-21

	2015-16		2019-21		Absolute difference between 2019-21 and 2015-16	
**Intervention**	**Non-priority states (N=60161)**	**Priority states (N=109284)**	**Non-priority states (N=60576)**	**Priority states (N=100573)**	**Non-priority states**	**Priority states**
**Pre-conception**						
Demand for FP satisfied	60.0	48.3***	60.5	58.4***	0.5	10.1
Iodized salt	95.8	92.5***	96.3	93.5***	0.5	1.0
**Pregnancy**						
Any ANC	87.7	77.1***	88.1	84.2***	0.4	7.1
ANC first trimester	62.7	54.1***	69.4	61.9***	6.7	7.7
≥4 ANC	64.2	45***	65.5	50.9***	1.3	5.9
Received MCP card	94.0	87.9***	97.2	95.6***	3.2	7.7
Received IFA tab/syrup	89.3	74.4***	94.1	85.7***	4.7	11.3
Neonatal tetanus	92.7	87.8***	92.9	91***	0.2	3.2
Deworming	19.2	18.2***	28.1	32.5***	8.9	14.3
Weighing	90.2	71.5***	95.0	89.9***	4.8	18.5
Breastfeeding counselling	52.6	36.8***	68.5	60.5***	15.9	23.7
Counselling on keeping baby warm	50.7	34.3***	66.7	57.8***	16.0	23.5
Cord care counselling	46.7	32.9***	63.8	56.7***	17.0	23.8
Food supplementation	62.7	50.7***	75.2	65.2***	12.5	14.5
Health & nutrition education	49.6	37.2***	64.7	59.1***	15.1	21.9
Use of bed nets	67.6	49.8***	67.7	52.5***	0.1	2.7
**Delivery & postnatal**						
Institutional birth	83.2	81.2***	92.5	89.3***	9.3	8.2
Skilled birth attendant	86.3	83.1***	93.1	89.8***	6.9	6.7
Food supplementation	58.5	46.5***	71.3	62.4***	12.8	15.8
Health & nutrition education	45.5	33.4***	60.9	55.6***	15.3	22.3
**Early childhood**						
Full immunization	74.2	59***	81.3	74.9***	7.1	15.9
Vitamin A	68.3	60.9***	66.8	68.1***	-1.5	7.2
Pediatric IFA	28.2	26***	35.9	38.9***	7.7	12.9
Deworming	38.9	30.8***	43.9	42.1***	5.0	11.2
Care seeking for ARI	86.8	86.6	60.9	56.4***	-25.9	-30.1
ORS during diarrhea	61.6	47.7***	65.6	58.7***	4.0	10.9
Zinc during diarrhea	22.7	19.3***	33.8	29.5***	11.1	10.1
Food supplementation	69.0	52.6***	78.9	68.6***	9.9	16.0
Weighing	56.2	41.8***	66.1	59.6***	9.9	17.8
Counselling on child growth	37.3	26.4***	47.6	45.4***	10.3	19.0

Notes: *p<0.05, **p<0.01, ***p < 0.001; All priority and non-priority estimates are significantly different (p<0.05) between 2016 and 2021 using the two-sample t test; priority states: under the national nutrition mission, 11 states were designated as priority states by the Indian government, and they received additional performance-based incentives to strengthen growth monitoring, home visits, and fixed-day services.

Supplemental Table 5: Interventions that were prioritized or received focus from national programs

Interventions	2016	2017	2018	2019	2020	2021
**Preconception**						
Demand for FP satisfied	Y	Y	NNM	NNM	NNM	NNM
Iodized salt	-	-	-	-	-	-
**Pregnancy**						
Any ANC	PMSMA	PMSMA<br>PMMVY	PMSMA<br>PMMVY<br>NNM	PMSMA<br>PMMVY<br>NNM	PMSMA<br>PMMVY<br>NNM	PMSMA<br>PMMVY<br>NNM
ANC first trimester	-	-	-	-	-	-
≥4 ANC	Y	Y	NNM	PMSMA<br>NNM	PMSMA<br>NNM	PMSMA<br>NNM
Received MCP card	PMSMA	PMSMA	PMSMA<br>PMMVY<br>NNM	PMSMA<br>PMMVY<br>HBYC<br>NNM	PMSMA<br>PMMVY<br>HBYC<br>NNM	PMSMA<br>PMMVY<br>HBYC<br>NNM
Received IFA tab/syrup	NIPI<br>PMSMA	PMSMA	NNM<br>AMB<br>PMSMA	NNM<br>AMB<br>PMSMA	NNM<br>AMB<br>PMSMA	NNM<br>AMB<br>PMSMA
Neonatal tetanus	Y	Y	NNM	NNM	NNM	NNM
Deworming	Y	Y	NNM<br>AMB	NNM<br>AMB	NNM<br>AMB	NNM<br>AMB
Weighing	PMSMA	PMSMA	PMSMA<br>NNM	PMSMA<br>NNM	PMSMA<br>NNM	PMSMA<br>NNM
Breastfeeding counselling	PMSMA	PMSMA	PMSMA<br>NNM	PMSMA<br>NNM	PMSMA<br>NNM	PMSMA<br>NNM
Counselling on keeping baby warm	PMSMA	PMSMA	PMSMA	PMSMA	PMSMA	PMSMA
Cord care counselling	PMSMA	PMSMA	PMSMA	PMSMA	PMSMA	PMSMA
Food supplementation	ICDS	ICDS	ICDS<br>NNM	ICDS<br>NNM	ICDS<br>NNM	ICDS<br>NNM
Health & nutrition education	ICDS	ICDS	ICDS<br>NNM	ICDS<br>NNM	ICDS<br>NNM	ICDS<br>NNM
Use of bed nets	Y	Y	NNM	NNM	NNM	NNM
**Delivery and postnatal care**						
Institutional birth	Y	Y	NNM	NNM	NNM	NNM
Skilled birth attendant	-	-	-	-	-	-
Food supplementation	ICDS	ICDS	ICDS<br>NNM	ICDS<br>NNM	ICDS<br>NNM	ICDS<br>NNM
Health & nutrition education	ICDS	ICDS	ICDS<br>NNM	ICDS<br>NNM	ICDS<br>NNM	ICDS<br>NNM
**Early childhood**						

Interventions	2016	2017	2018	2019	2020	2021
Full immunization	Y	PMMVY	PMMVY<br>NNM	PMMVY<br>HBYC<br>NNM	PMMVY<br>HBYC<br>NNM	PMMVY<br>HBYC<br>NNM
Vitamin A	Y	Y	NNM	NNM	NNM	NNM
Pediatric IFA	Y	Y	AMB<br>NNM	AMB<br>HBYC<br>NNM	AMB<br>HBYC<br>NNM	AMB<br>HBYC<br>NNM
Deworming	Y	Y	AMB<br>NNM	AMB<br>HBYC<br>NNM	AMB<br>HBYC<br>NNM	AMB<br>HBYC<br>NNM
Care seeking for ARI	Y	Y	NNM	NNM	NNM	NNM
ORS during diarrhea	Y	Y	NNM	HBYC<br>NNM	HBYC<br>NNM	HBYC<br>NNM
Zinc during diarrhea	Y	Y	NNM	HBYC<br>NNM	HBYC<br>NNM	HBYC<br>NNM
Food supplementation	ICDS	ICDS	ICDS<br>NNM	ICDS<br>HBYC<br>NNM	ICDS<br>HBYC<br>NNM	ICDS<br>HBYC<br>NNM
Weighing	ICDS	ICDS	ICDS<br>NNM	ICDS<br>HBYC<br>NNM	ICDS<br>HBYC<br>NNM	ICDS<br>HBYC<br>NNM
Counselling on child growth	ICDS	ICDS	ICDS<br>NNM	ICDS<br>HBYC<br>NNM	ICDS<br>HBYC<br>NNM	ICDS<br>HBYC<br>NNM

NNM: National Nutrition Mission; PMSMA: Pradhan Mantri Surakshit Matritva Abhiyan; PMMVY: Pradhan Mantri Matru Vandana Yojana; HBYC: Home Based Care for Young Children; AMB: Anemia Mukt Bharat; Y: Included as part of ICDS or Health programs; FP: Family planning ANC: Antenatal care MCP: Mother child protection IFA: Iron folic acid CHW: Community health worker ICDS: Integrated Child Development Scheme NNM: National Nutrition Mission

Supplemental Figure 4: Annual average rate of increase in coverage of interventions, by priority and non-priority states between 2005-06 and 2015-16 and 2019-21

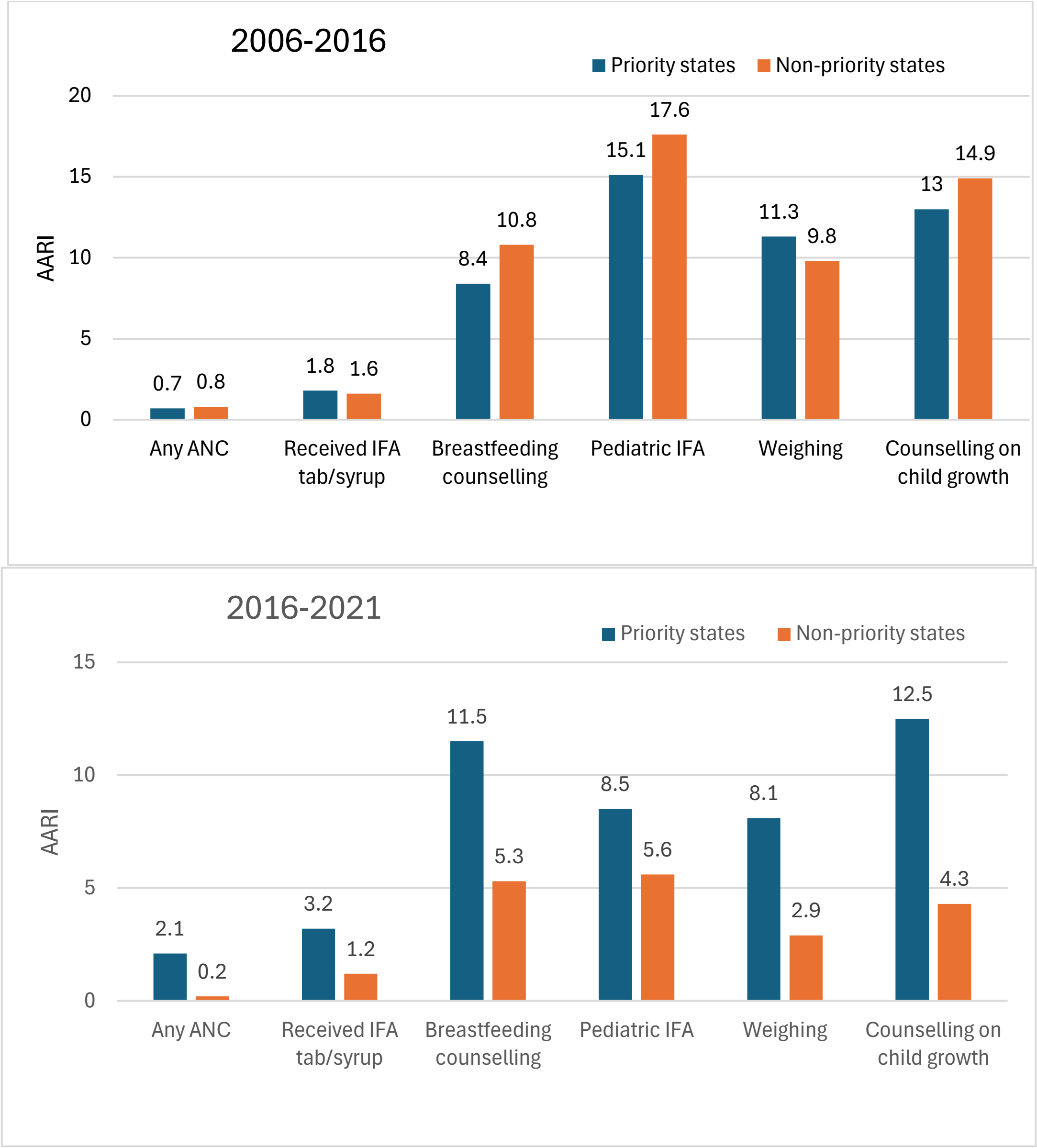

Note: AARI: Annual Average Rate of Increase

Web appendix 1: Construction of wealth index and quintiles

We have used household-owned assets and amenities to construct the wealth index using data on a household's characteristics: source of drinking water, type of toilet facilities, type of flooring, exterior wall material, type of roofing, cooking fuel, electricity, home ownership, domestic helper, number of household members per sleeping room, ownership of a bank or post office account and having a mattress, pressure cooker, chair, cot/bed, table, electric fan, radio/transistor, black-and-white television, color television, sewing machine, mobile phone ,any other telephone, internet , computer, refrigerator, air cooler ,washing machine, watch or clock, bicycle, motorcycle or scooter, an animal-drawn cart, car, water pump, thresher, tractor and livestock(cows ,camels, goats, horse,chicken,pigs).
